# Prenatal sex determination illuminates the unusual adult sex ratio of a group-living lemur

**DOI:** 10.1098/rsbl.2024.0418

**Published:** 2025-02-26

**Authors:** Leonie Pethig, Arpat Ozgul, Michael Heistermann, Claudia Fichtel, Peter M. Kappeler

**Affiliations:** ^1^ Behavioral Ecology and Sociobiology Unit, German Primate Center, Leibniz Institute for Primate Research, Göttingen 37077, Germany; ^2^ Department of Sociobiology/Anthropology, Johann-Friedrich-Blumenbach Institute of Zoology and Anthropology, University of Göttingen, Göttingen 37077, Germany; ^3^ Department of Evolutionary Biology and Environmental Studies, University of Zürich, Zürich 8057, Switzerland; ^4^ Endocrinology Laboratory, German Primate Center, Leibniz Institute for Primate Research, Göttingen 37077, Germany

**Keywords:** sex ratios, lemurs, prenatal sex determination, survival, reproductive costs

## Abstract

Most mammals, including humans, exhibit even or slightly male-biased birth sex ratios (BSRs) and female-biased adult sex ratios (ASRs) much later in life due to higher male mortality rates. The group-living primates of Madagascar are unusual in this respect because they lack female-biased ASRs, but it is unknown whether this is the result of skewed BSRs or sex-specific disappearance patterns. Using long-term demographic data from wild red-fronted lemurs (*Eulemur rufifrons*), we analysed their sex ratio dynamics across the lifespan. We assessed BSR via prenatal sex determination using maternal faecal oestrogen metabolite measurements during late pregnancy, confirming a visually determined equal sex ratio three months after birth, and indicating no early sex-specific mortality. Demographic analyses additionally disclosed higher female disappearance within the first 8 years of age, likely associated with reproductive effort early in life. Thereby, adult male survival had the greatest positive effect on the ASR. Our study offers a rare perspective on the dynamics of age- and sex-specific disappearance in a wild primate population, whose sex-reversed patterns may also contribute to a more general understanding of the mechanisms generating sex-biased mortality.

## 1. Introduction

The adult sex ratio (ASR), i.e. the proportion of adult males to females, is highly variable across species, within populations and across age classes, highlighting the complex interplay of multiple biological adaptations [1]. While understanding the causes and dynamics of sex biases in ASR remains a core problem in animal ecology [2,3] and sexual selection research [4–8], sex ratios have also become a key topic in human biology and medicine [9,10]. In humans, about 107 boys are born on average per every 100 girls [11], but the list of the 100 oldest verified living people includes 96 women and only four men in 2024 [12], indicating massive shifts in the human sex ratio across the lifespan. Notably, female human life expectancy considerably exceeds that of males worldwide [13,14], even though women spend a larger proportion of their life expectancy in poorer health [15,16]. As a result, men beyond the age of around 50 are outnumbered by women [17,18], reversing the original sex ratio bias at birth ([19]; but see [20,21]).

Studies of other primates and mammals, which share fundamental features of their life history with humans, can contribute important comparative information on the nature, drivers and relative importance of sex differences in mortality shaping prevailing sex ratios [22–24]. However, relevant studies of age- and sex-related variation in survival have been largely limited to laboratory rodents ([22]; but see [25]). Notable exceptions are provided by several studies of birds (e.g. [3,26–28]) and savannah baboons (*Papio cynocephalus*), where females enjoy greater longevity than males, but unlike humans, the declines in several measures of health with age are the same for both sexes or even more pronounced in males [29]. Other studies focusing on aspects of age-related sex differences in mortality or specific components of health and survival also indicate that male mammals generally suffer greater mortality than females (primates [30]; red deer [31,32]; European badgers [33]; grey mouse lemurs [34,35]; chimpanzees [36,37]; bottlenose dolphins [38]; lions [39]). Contrastingly, in meerkats (*Suricata suricatta*), both sexes displayed similar rates of age-related survival and body mass senescence [40].

A comparative study revealed that males had higher age-specific mortality than females in six out of seven primate species [30]. In addition, a meta-analysis indicates that the median female lifespan among 101 mammal species is on average about 20% longer than in males, even though the sexes experience similar increasing rates in mortality risk with age [23]. However, both variables were highly erratic across species (see also [41]) and responsive to changes in environmental conditions [42]. Thus, sex differences in survival clearly appear more variable across species of wild mammals than the pattern reported for humans, but a male bias in adult mortality seems to be widespread and robust [30,43–45]. As a result, there are very few males alive in the oldest age group of most mammalian populations, contributing to on average female-biased ASRs [46–50].

Given this context, the absence of female-biased ASRs among group-living primates of Madagascar (Lemuriformes) remains an intriguing anomaly compared with most other non-monogamous mammals [46,51,52]. Because lemurs also lack sexual size dimorphism [53,54], the energetic costs of growing larger bodies do not accrue for lemur males. Lemurs are further characterized by widespread female dominance and genital masculinization [55–57], indicating intense female competition [58], but the ultimate causes and dynamics of the unusual ASRs in lemurs remain obscure. Theoretically, it is possible that birth sex ratios (BSRs) are male-biased, but in a large captive lemur colony, BSRs did not differ significantly from 1 : 1 in 18 out of 19 species [59], whereas they were male-biased in all six species included in an earlier comparative study [60]. However, because BSRs in lemurs and other primates can be adapted to variation in social and environmental factors [61–64], data from wild populations are ultimately required. Furthermore, virtually all currently available data on sex ratios of wild lemurs are based on cross-sectional reports of average group compositions or studies of short duration, or stem from populations subject to poaching [65–71]. In this study, we present the first long-term data on sex ratio dynamics of a wild lemur population, filling a significant gap in the current sex ratio literature.

We have been studying a population of individually marked red-fronted lemurs (*Eulemur rufifrons*) in Kirindy Forest since 1996. In this sexually dichromatic species, the sex of infants can only be determined visually at about three months, when the coats of female infants change from the male to the female phenotype [72]. In the first 7 years of our long-term study, the ASR was male-biased and the infant sex ratio at 12 weeks was even, but age- and sex-specific disappearance could not be determined with the sample size available at the time [73]. Their BSR remains unknown, but we discovered that maternal oestrogen levels during late gestation vary as a function of infant sex [74], offering an opportunity to non-invasively determine a key milestone in sex ratio dynamics and to exclude early-life mortality during the first three months in this population. Here, we combine demographic data on 302 wild red-fronted lemurs with non-invasively collected hormone samples to determine their BSRs and age-specific disappearance rates, including death and dispersal and eviction events, of both sexes across the lifespan. To explain the male-biased ASR, we explore two hypotheses: one posits strongly male-biased BSRs followed by typical mammalian post-natal disappearance patterns; the other suggests even BSRs with subsequent higher disappearance rates for juvenile and/or adult females.

## 2. Material and methods

### (a) Study population

Red-fronted lemurs live in small cohesive groups (mean 2.6 adult females and 3.3 adult males) with on average male-biased ASRs [51,73]. Despite similar body mass between males (1490 ± 400 g (mean ± s.d.), *n* = 178) and females (1501 ± 412 g (mean ± s.d.), *n* = 132; *t*‐test: t = 0.24, d.f. = 275.56, *p* = 0.81), they exhibit striking sexual dichromatism (electronic supplementary material, figure S1). Sexual maturity is attained in the third year of life. Philopatric females usually produce single infants annually thereafter, which they exclusively care for, as no paternal, communal or cooperative caregiving has been observed in this species. On average, females give birth to 4.83 ± 3.52 (mean ± s.d.) infants during their lifetime, with the first reproduction occurring at 3.59 ± 0.82 years (mean ± s.d.) and a decline in birth probability after 8.22 ± 4.22 years (mean ± s.d.) [75]. Maximum lifespan in the wild is about 25 years, while in captivity, males and females can live up to 32.1 years and 32.6 years, respectively [59].

Data for this study are based on 27 years of demographic census observations of a population of red-fronted lemurs in Kirindy Forest, western Madagascar. Since 1996, all members of seven groups have been individually marked with RFID transponders and unique nylon or radio collars [76], facilitating near-daily censuses, during which the composition of each group was recorded. For all immigrants not born into our study groups, we estimated ages based on tooth wear and body mass. Apart from births and deaths, group sex ratios are modified by male dispersal (emigration/immigration) and female evictions [58,76]. Such events were recorded if an individual was absent for a minimum of four consecutive weeks, re-sighted in another group or remains were discovered.

### (b) Prenatal sex ratio determination

**Figure 1.**
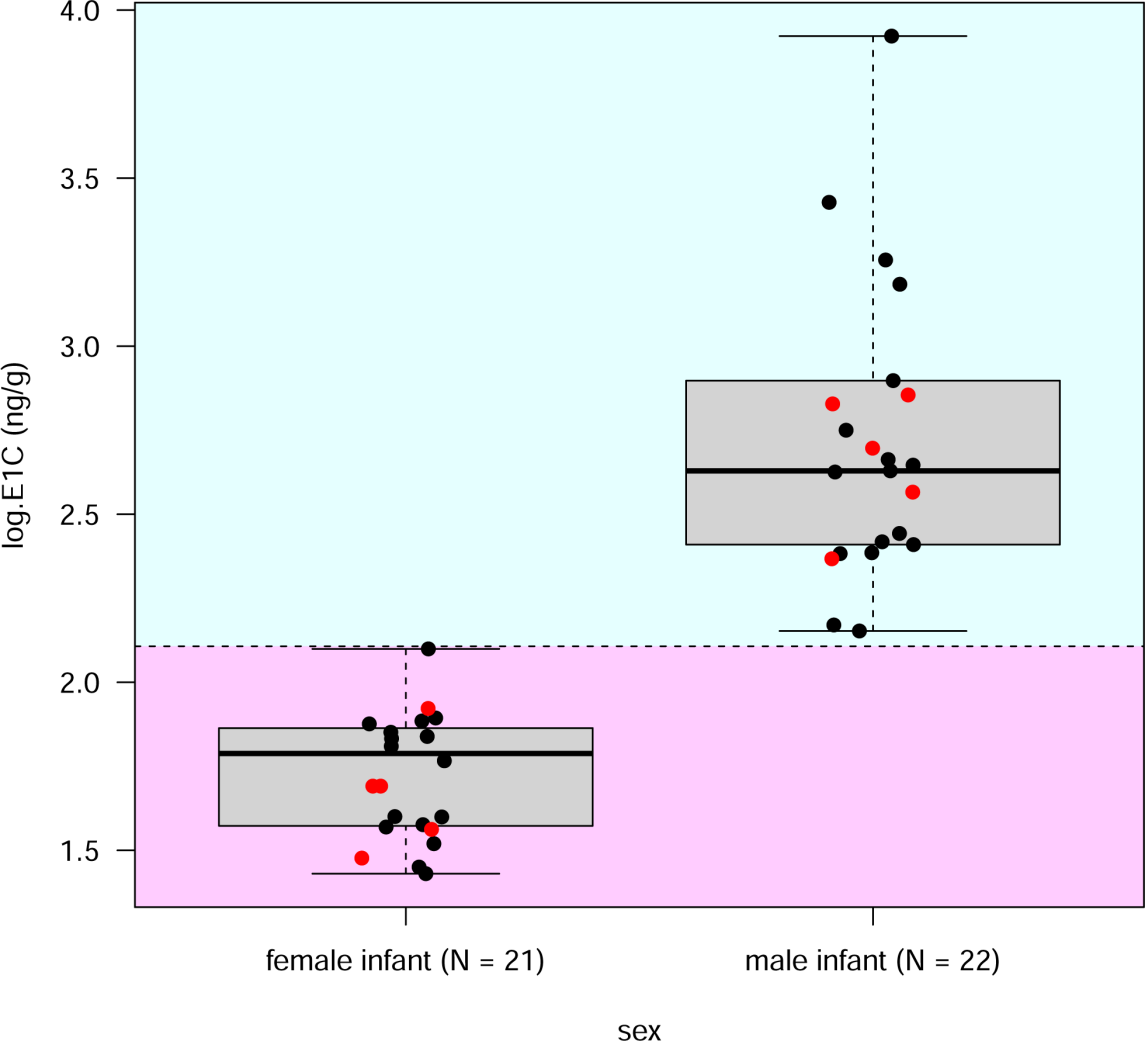
Foetal sex and maternal faecal oestrogen (E1C) concentrations in red-fronted lemurs. Box plots include visually confirmed pregnancies (black circles) and hormonally confirmed pregnancies (red circles), where births have not been observed. The horizontal dashed line indicates a threshold at 2 s.d., separating mean oestrogen values of mothers with female infants (pink background) from those carrying male infants (blue background).

Because all infants are born with the male phenotype (electronic supplementary material, figure S1), it only becomes possible to visually distinguish male from female infants at about 12 weeks of age [72]. Yet, infant mortality can be high [77], and the loss of an offspring of one or the other sex might bias the BSR value determined by visual inspections and thus the starting point for subsequent survival analyses. To accurately assess BSRs and potential sex-specific mortality within the first three months, we determined offspring sex prenatally based on maternal excreted oestrogen metabolite measurements during late pregnancy [74,78,79]. Faecal samples for oestrogen metabolite analyses were available from 2015 until 2022 and were measured for concentrations of immunoreactive oestrone, the major faecal oestrogen in the red-fronted lemur [74], using an enzyme immunoassay for oestrone conjugates (E1C) as described in [80]. We re-validated the method (electronic supplementary material, Method re-validation) and foetal sex could be assessed with 100% accuracy because only mothers with a male offspring had markedly elevated oestrogen levels during the last six weeks of gestation (figure 1). We combined individual numbers of male and female offspring generated from the demographic and hormonal data sets and calculated the BSR for this population using the following equation:


(2.1) $$BSR=\frac{\left(N_{male\;offspring}\right)}{\left(N_{male\;offspring}\;+N_{female\;offspring}\right)}$$

Confidence intervals for BSR were obtained using bootstrap resampling, applying 1000 parametric bootstraps.

### (c) Event-history analysis

A population overview revealed that a skewed sex ratio is only present in the adult stage (≥ 30 months of age), with higher numbers of males across almost all 27 years (electronic supplementary material, figure S2). Using a data set, including visually and hormonally determined infant sexes, we fitted a Cox proportional hazards (CPH) model, using the *survival* [81] and *survminer* [82] packages in R (v. 4.3.0; [83]), to examine whether the probability of staying in a group is predicted by sex. Individual histories ending with the final census date were assigned 0, i.e. right-censoring.

### (d) Population projection model

To evaluate how changes in individual matrix elements influence the ASR, a numerical sensitivity analysis was conducted. We parameterized a population projection model using the Kaplan–Meier method to derive average survival rates, calculated across all years. Mean annual fecundity rates were calculated for female and male offspring separately. These rates informed a Leslie matrix with four age-sex classes, providing insights into the relative impact of each demographic process (e.g. survival, reproduction) on the ASR. Details of the model structure, parameterization and calculations are provided in the electronic supplementary material, Demographic additions.

## 3. Results

### (a) Life-history data

Over 27 years, we recorded 193 births (83 females, 74 males, 36 unknown sexes) by 41 different females. In total, 266 individuals disappeared, either due to confirmed death (13 females, 13 males, two unknown sexes; *n* = 28), confirmed male emigration (*n* = 78), confirmed female eviction (*n* = 32) or unknown causes (58 females, 70 males; *n* = 128). The oldest male and female in this study population reached an age of 17 and 23 years, respectively.

### (b) Prenatal sex ratio determination

Between 2015 and 2022, we analysed faecal hormone data of 20 females and detected 43 pregnancies. We prenatally determined 21 female and 22 male fetuses (figure 1), with an average sex ratio of 0.51 (after equation (2.1)). Combining the numbers of visually and hormonally determined female (*n* = 88) and male (*n* = 79) offspring from 201 confirmed pregnancies (34 unknown sexes), we obtained a BSR of 0.47, with a 95% confidence interval ranging from 0.38 to 0.54 (electronic supplementary material, table S4).

### (c) Demographic analyses

**Figure 2.**
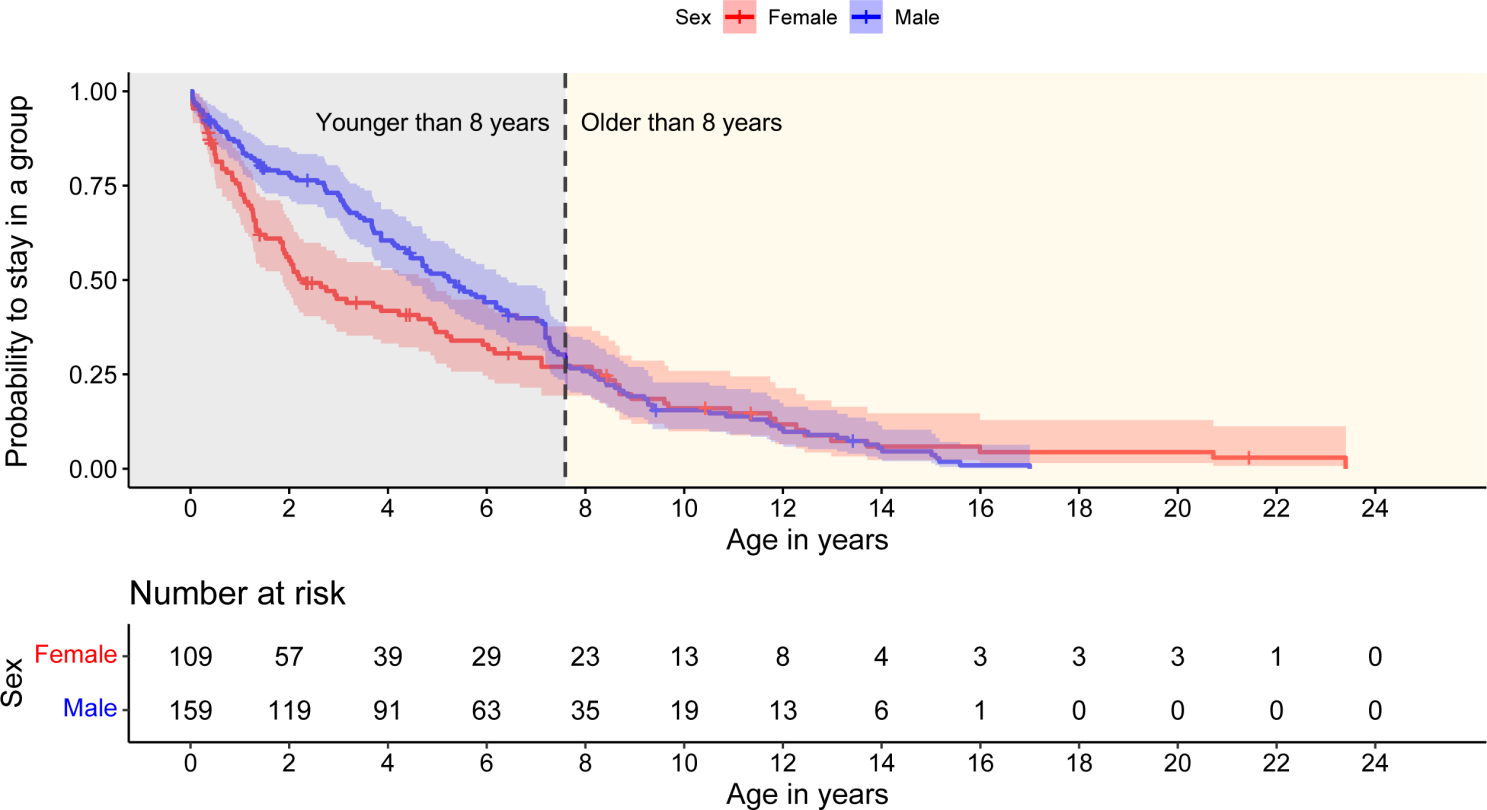
Sex- and age-specific disappearances of red-fronted lemurs. Males have a higher probability of staying in a group until about 8 years of age. ‘Number at risk’ indicates the number of individuals who have not yet experienced the event of disappearance.

Censuses between 1996 and 2023 yielded life-history data of 159 males (79 natal, 80 immigrants) and 109 females for this study. A total of 34 individuals disappeared before they could be sexed, indicating that about 20% of offspring likely died within the first three months of age. Individuals that left a group were characterized as ‘1’ despite successful dispersal because we were mainly interested in whether sex predicts individual disappearance. Overall, sex did not predict individual disappearance (likelihood ratio test = *X*^2^ = 1.2*,* d.f. = 1, *p* = 0.3, sex (male): coef = −0.2, s.e. = 0.14, *p* = 0.267), but hazards were not proportional (sex: *X*^2^ = 14.6, d.f. = 1, *p* < 0.001, global: *X*^2^ = 14.6, d.f. = 1, *p* < 0.001). As there was a sex-specific change in the probability of staying in a group below and above an age of 8 years (figure 2), we included an interaction between sex and a term consisting of two levels, i.e. ‘<8 years’ and ‘≥8 years’, resulting in proportional hazards for all terms (sex: *X*^2^ = 0.1, d.f. = 1, *p* = 0.75, <8 years: *X*^2^ = 0.08, d.f. = 1, *p* = 0.77, sex: <8 years: *X*^2^ = 0.02, d.f. = 1, *p* = 0.88, global: *X*^2^ = 0.9, d.f. = 3, *p* = 0.83). This CPH analysis indicated that, during the first 8 years of life, males were more likely to remain in a group compared with females (*p* = 0.002; Wald test = 52.77, d.f. = 3, *p* < 0.001; likelihood ratio test: *X*^2^ = 254.3*,* d.f. = 3, *p* < 0.001; table 1; figure 2), even though male natal dispersal usually begins in the third year of life [73]. At around 8 years of age, male and female disappearance rates were about equal.

**Table 1.** Output of the CPH model. Males younger than 8 years have a lower probability of disappearing from a group.

term	coefficient	s.e.	*p*
sex (male)	0.33	0.30	0.282
younger than 8 years	6.59	1.06	<0.001***
sex(male): younger than 8 years	−1.06	0.34	0.002**

Significant effects in bold. Significance codes: 0 ‘***’ 0.001 ‘**’ 0.01 ‘*’ 0.05 ‘.’ 0.1 ‘ ’ 1

The sensitivity analysis revealed that adult male survival had the greatest positive effect on the ASR, with increases in these rates significantly raising the proportion of males in the population (electronic supplementary material, Results and table S3), resulting in a moderately male-biased ASR (0.61; see also electronic supplementary material, table S4).

## 4. Discussion

Our study of a wild population of red-fronted lemurs not only confirmed the existence of an even sex ratio at birth but also found that, unlike in most non-monogamous mammals [44], the BSR shifted towards male-biased ASRs due to higher female disappearances within the first 8 years of life. We were able to pinpoint this crucial time window and the direction of the subsequent sex difference in disappearance for understanding the unusual lemur ASR because we assessed early infant mortality via hormonal prenatal sex determination. The hormonally and visually determined foetal and infant sex ratios revealed a congruent lack of sex bias and provided a reliable baseline for subsequent analyses of their sex ratio dynamics. In this study, BSRs had to be established as a starting point for the following demographic analyses because of high early mortality of yet unsexed infants. Thus, the prenatally determined sex ratio, the even BSR and the subsequent female disappearance bias are new key pieces of the lemur sex ratio dynamics puzzle.

Prior to this study, comprehensive sex ratio data were available for three members of the Indriidae family. In *Propithecus verreauxi*, BSR was apparently heavily male-biased, but infants have only been sexed as yearlings [66]. Later on, young adult females experienced higher mortality than males, but this pattern reversed around age 12 [66]. In *P. edwardsi*, the sex ratio was female-biased at birth, became male-biased at about 3 years and female-biased again from age 18 onwards [70]. Between the ages of 2 and 18 years, males enjoyed higher survival than females, but this sex difference quickly reversed afterwards. However, sample sizes for older adults were small, and the ages of most males in the study were estimated. In *Indri indri*, the sex ratio of yearlings was female-biased, and life expectancy at age 2 was similar in both sexes, but males suffered from marginally higher age-independent mortality [71]. For the Lemuridae, the other family with group-living taxa, only preliminary age- and sex-specific demographic data are available for four of the 21 species. In *Lemur catta* and *E. flavifrons*, data were either not sex-specific [65,69] or limited to females [84]. In *E. macaco*, sex ratios were male-biased at birth, for juveniles and for adults, with males having higher survival rates, but this population was subject to poaching and only studied for seven months [67]. In another *E. rufifrons* population, BSR and infant survival were male-biased, but sex-specific mortality remained unknown for later ages [68]. Thus, previous data could not identify a uniform pattern in sex ratio dynamics of group-living lemurs.

Sexing lemur infants at birth is hampered by two main problems. First, in some genera (*Lemur*, *Hapalemur*, *Prolemur*, *Varecia*, *Indri*), males and females have sexually monomorphic coats and—because of an enlarged clitoris—cannot be reliably distinguished visually at young ages. Second, nine of the 12 *Eulemur* species are sexually dichromatic as adults, but infants are born with the same coat coloration [72,85], also obstructing visual sexing during the first months of life. As early infant mortality in lemurs is generally high [77], sex ratio estimates later in infancy might be misleading, especially if early mortality is sex-biased [38,60,86]. Sexing by physiological instead of visual methods is therefore essential for accurate assessments of BSRs. While this can be achieved by DNA analysis [87], such samples are usually difficult to obtain from infants. Moreover, since DNA sampling may not be possible in cases of abortions, stillbirths or very early infant disappearances, this method has limited reliability for determining a potential sex bias in early life mortality, an important aspect when studying sex ratio dynamics in wild animal populations. To overcome these caveats, we assessed offspring sex prenatally through non-invasive analyses of maternal hormone concentrations during gestation [74,78,79,88–90]. To our knowledge, our study is the first to utilize this approach to assess BSRs and examine the degree of sex-specificity in early life mortality at a population level, highlighting its potential for non-invasively assessing BSRs and pregnancy failure in wild mammals, once species-specific endocrine correlates have been established.

Solid evidence for an even BSR and absence of sex-specific mortality very early in life allowed us to explore the subsequent sex ratio dynamics. In stark contrast to the pattern characterizing the vast majority of polygynous mammals, our analyses revealed higher male survival rates that inherently imply an increased risk for females to disappear in the first 8 years of life, driving the observed male bias in ASR. Previous studies on lemur sex ratios and sexual monomorphism have focused on alternative mechanisms of male competition to explain the apparent lack of increased adult male mortality [55,91,92] because sexual selection theory offers an evolutionary framework for analysing adaptive sex differences in health and mortality. Accordingly, male, but not female, reproductive success in mammals is thought to be limited by mating access, generating a trade-off between reproduction and maintenance [43]. In particular, investment in reproduction early in life will have detrimental effects on somatic maintenance later in life [93]. The fact that female competition is widespread and can have similar evolutionary effects has only recently been acknowledged, however [94].

Specifically, studies of a few species that also deviate from this typical mammalian pattern (*Marmota marmota* [95]; *S. suricatta* [40]; *Equus ferus caballus* [96]) helped to refine the sexual selection hypothesis for explaining sex biases in ASR. They demonstrated that appreciation of selection pressures generated by intrasexual competition in both sexes offers better explanations for sex-biased mortality than crude mating system categories (see also [45]). Female reproduction in this red-fronted lemur population declined indeed massively after an average of 8 years [75], coinciding with the onset of more balanced disappearance probabilities among the sexes. Investment in intrasexual reproductive competition entails costs to somatic maintenance, leading to higher rates of senescence in the sex experiencing stronger reproductive competition, as has been demonstrated in European badgers (*Meles meles* [97]). In addition, the mortality risk of red-fronted and some other lemur females is elevated by eviction, an extreme form of intrasexual competition, in which females are forced to leave their natal group [58,76]. As in bottlenose dolphins (*Tursiops aduncus*), these costs of female reproduction, including sexual coercion and competition, may be so high that they affect sex ratios across the lifespan, closing or even reversing sex gaps in mortality hazards [38].

By illuminating the mechanisms driving sex-specific patterns in wild mammal species, we can obtain insights into the adaptive strategies of both sexes, sex-specific causes of disappearance and sex ratio dynamics across the complete lifespan. Such a comparative approach may also contribute to a broader understanding of sex-specific mortality patterns in other mammals. We know most about the underlying mechanisms in humans, where female reproductive effort is also costly [98], but other proximate drivers, including immune system responses, hormonal effects and cellular senescence, as well as risk-taking behaviours and unhealthy lifestyles [15,24], may outweigh these costs. Our findings therefore underscore the significance of understanding species- and sex-specific causes of disappearance or mortality to broaden our comprehension of demographic and evolutionary processes related to mortality patterns.

## Data Availability

Data, R codes and a README file to conduct all analyses are available from Figshare [99]. Additional electronic supplementary material, figures S1, S2 and tables S1–S4, and supporting information to the methods section are available online [100].

## References

[1] Schacht R *et al*. 2022 Adult sex ratios: causes of variation and implications for animal and human societies. Commun. Biol. **5**, 1273. (10.1038/s42003-022-04223-w) 36402823 PMC9675760

[2] Ancona S, Dénes FV, Krüger O, Székely T, Beissinger SR. 2017 Estimating adult sex ratios in nature. Phil. Trans. R. Soc. B **372**, 20160313. (10.1098/rstb.2016.0313) 28760756 PMC5540855

[3] Székely T, Liker A, Freckleton RP, Fichtel C, Kappeler PM. 2014 Sex-biased survival predicts adult sex ratio variation in wild birds. Proc. R. Soc. B **281**, 20140342. (10.1098/rspb.2014.0342) PMC4083783 24966308

[4] Kokko H, Jennions MD. 2008 Parental investment, sexual selection and sex ratios. J. Evol. Biol. **21**, 919–948. (10.1111/j.1420-9101.2008.01540.x) 18462318

[5] Székely T, Weissing FJ, Komdeur J. 2014 Adult sex ratio variation: implications for breeding system evolution. J. Evol. Biol. **27**, 1500–1512. (10.1111/jeb.12415) 24848871

[6] Janicke T, Häderer IK, Lajeunesse MJ, Anthes N. 2016 Darwinian sex roles confirmed across the animal kingdom. Sci. Adv. **2**, e1500983. (10.1126/sciadv.1500983) 26933680 PMC4758741

[7] Janicke T, Morrow EH. 2018 Operational sex ratio predicts the opportunity and direction of sexual selection across animals. Ecol. Lett. **21**, 384–391. (10.1111/ele.12907) 29341415

[8] Kappeler PM *et al*. 2022 Sex roles and sex ratios in animals. Biol. Rev. **98**, 462–480. (10.1111/brv.12915) 36307924

[9] Lazarus J. 2002 Human sex ratios: adaptations and mechanisms, problems and prospects. In Sex ratios: concepts and research methods (ed. I Hardy), pp. 287–311. Cambridge, UK: Cambridge University Press. (10.1017/CBO9780511542053.015)

[10] Legato MJ. 2023 Principles of gender-specific medicine: sex and gender-specific biology in the postgenomic era., 4th edn. Oxford, UK: Academic Press.

[11] Chao F, Gerland P, Cook AR, Alkema L. 2019 Systematic assessment of the sex ratio at birth for all countries and estimation of national imbalances and regional reference levels. Proc. Natl Acad. Sci. USA **116**, 9303–9311. (10.1073/pnas.1812593116) 30988199 PMC6511063

[12] Wikipedia. The Free Encyclopedia. See https://en.wikipedia.org/wiki/List_of_the_verified_oldest_people (accessed 13 December 2024).

[13] Barford A, Dorling D, Smith GD, Shaw M. 2006 Life expectancy: women now on top everywhere. BMJ **332**, 808. (10.1136/bmj.332.7545.808) 16601021 PMC1432200

[14] Thorslund M, Wastesson JW, Agahi N, Lagergren M, Parker MG. 2013 The rise and fall of women’s advantage: a comparison of national trends in life expectancy at age 65 years. Eur. J. Ageing **10**, 271–277. (10.1007/s10433-013-0274-8) 24319404 PMC3851807

[15] Oksuzyan A, Juel K, Vaupel JW, Christensen K. 2008 Men: good health and high mortality. Sex differences in health and aging. Aging Clin. Exp. Res. **20**, 91–102. (10.1007/bf03324754) 18431075 PMC3629373

[16] Hossin MZ. 2021 The male disadvantage in life expectancy: can we close the gender gap? Int. Health **13**, 482–484. (10.1093/inthealth/ihaa106) 33533409 PMC7928849

[17] Beltrán-Sánchez H, Finch CE, Crimmins EM. 2015 Twentieth century surge of excess adult male mortality. Proc. Natl Acad. Sci. USA **112**, 8993–8998. (10.1073/pnas.1421942112) 26150507 PMC4517277

[18] Hollingshaus M, Utz R, Schacht R, Smith KR. 2019 Sex ratios and life tables: historical demography of the age at which women outnumber men in seven countries, 1850–2016. Hist. Methods **52**, 244–253. (10.1080/01615440.2019.1605863)

[19] Ritchie H, Roser M. 2019 Gender ratio: how does the number of men and women differ between countries? And why? Our World in Data. See https://ourworldindata.org/gender-ratio.

[20] Austad SN. 2015 The human prenatal sex ratio: a major surprise. Proc. Natl Acad. Sci. USA **112**, 4839–4840. (10.1073/pnas.1505165112) 25848060 PMC4413335

[21] Orzack SH, Stubblefield JW, Akmaev VR, Colls P, Munné S, Scholl T, Steinsaltz D, Zuckerman JE. 2015 The human sex ratio from conception to birth. Proc. Natl Acad. Sci. USA **112**, E2102–11. (10.1073/pnas.1416546112) 25825766 PMC4413259

[22] Austad SN, Fischer KE. 2016 Sex differences in lifespan. Cell Metab. **23**, 1022–1033. (10.1016/j.cmet.2016.05.019) 27304504 PMC4932837

[23] Lemaître JF *et al*. 2020 Sex differences in adult lifespan and aging rates of mortality across wild mammals. Proc. Natl Acad. Sci. USA **117**, 8546–8553. (10.1073/pnas.1911999117) 32205429 PMC7165438

[24] Hägg S, Jylhävä J. 2021 Sex differences in biological aging with a focus on human studies. eLife **10**, e63425. (10.7554/elife.63425) 33982659 PMC8118651

[25] Larson SM, Colchero F, Jones OR, Williams L, Fernandez-Duque E. 2016 Age and sex-specific mortality of wild and captive populations of a monogamous pair-bonded primate (Aotus azarae). Am. J. Primatol. **78**, 315–325. (10.1002/ajp.22408) 25866126 PMC5611823

[26] Eberhart-Phillips LJ *et al*. 2017 Sex-specific early survival drives adult sex ratio bias in snowy plovers and impacts mating system and population growth. Proc. Natl Acad. Sci. USA **114**, E5474–E5481. (10.1073/pnas.1620043114) 28634289 PMC5502594

[27] Eberhart-Phillips LJ *et al*. 2018 Demographic causes of adult sex ratio variation and their consequences for parental cooperation. Nat. Commun. **9**, 1651. (10.1038/s41467-018-03833-5) 29695803 PMC5917032

[28] Donald PF. 2007 Adult sex ratios in wild bird populations. Ibis **149**, 671–692. (10.1111/j.1474-919x.2007.00724.x)

[29] Alberts SC, Archie EA, Gesquiere LR, Altmann J, Vaupel JW, Christensen K. 2014 The male–female health–survival paradox: a comparative perspective on sex differences in aging and mortality. In Sociality, hierarchy, health: comparative biodemography: a collection of papers (eds M Weinstein, MA Lane), pp. 339–363. Washington, DC: The National Academies Press. 25254285

[30] Bronikowski AM *et al*. 2011 Aging in the natural world: comparative data reveal similar mortality patterns across primates. Science **331**, 1325–1328. (10.1126/science.1201571) 21393544 PMC3396421

[31] Loison A, Festa-Bianchet M, Gaillard JM, Jorgenson JT, Jullien JM. 1999 Age-specific survival in five populations of ungulates: evidence of senescence. Ecology **80**, 2539–2554. (10.1890/0012-9658(1999)080[2539:assifp]2.0.co;2)

[32] Catchpole EA, Fan Y, Morgan BJT, Clutton-Brock TH, Coulson T. 2004 Sexual dimorphism, survival and dispersal in red deer. JABES **9**, 1–26. (10.1198/1085711043172))

[33] Sugianto NA, Newman C, Macdonald DW, Buesching CD. 2020 Reproductive and somatic senescence in the European badger (Meles meles): evidence from lifetime sex-steroid profiles. Zoology **141**, 125803. (10.1016/j.zool.2020.125803) 32574816

[34] Kraus C, Eberle M, Kappeler PM. 2008 The costs of risky male behaviour: sex differences in seasonal survival in a small sexually monomorphic primate. Proc. R. Soc. B **275**, 1635–1644. (10.1098/rspb.2008.0200) PMC2602817 18426751

[35] Ozgul A, Fichtel C, Paniw M, Kappeler PM. 2023 Destabilizing effect of climate change on the persistence of a short-lived primate. Proc. Natl Acad. Sci. USA **120**, e2214244120. (10.1073/pnas.2214244120) 36972440 PMC10083614

[36] Thompson ME, Machanda ZP, Fox SA, Sabbi KH, Otali E, Thompson González N, Muller MN, Wrangham RW. 2020 Evaluating the impact of physical frailty during ageing in wild chimpanzees (Pan troglodytes schweinfurthii). Phil. Trans. R. Soc. B **375**, 20190607. (10.1098/rstb.2019.0607) 32951544 PMC7540960

[37] Emery Thompson M *et al*. 2020 Wild chimpanzees exhibit humanlike aging of glucocorticoid regulation. Proc. Natl Acad. Sci. USA **117**, 8424–8430. (10.1073/pnas.1920593117) 32229565 PMC7165472

[38] McEntee MHF, Foroughirad V, Krzyszczyk E, Mann J. 2023 Sex bias in mortality risk changes over the lifespan of bottlenose dolphins. Proc. R. Soc. B **290**, 20230675. (10.1098/rspb.2023.0675) PMC10369037 37491966

[39] Barthold JA, Loveridge AJ, Macdonald DW, Packer C, Colchero F. 2016 Bayesian estimates of male and female African lion mortality for future use in population management. J. Appl. Ecol. **53**, 295–304. (10.1111/1365-2664.12594)

[40] Thorley J, Duncan C, Sharp SP, Gaynor D, Manser MB, Clutton‐Brock T. 2020 Sex‐independent senescence in a cooperatively breeding mammal. J. Anim. Ecol. **89**, 1080–1093. (10.1111/1365-2656.13173) 31943191

[41] Colchero F *et al*. 2016 The emergence of longevous populations. Proc. Natl Acad. Sci. USA **113**, E7681–E7690. (10.1073/pnas.1612191113) 27872299 PMC5137748

[42] Tidière M, Gaillard JM, Berger V, Müller DWH, Bingaman Lackey L, Gimenez O, Clauss M, Lemaître JF. 2016 Comparative analyses of longevity and senescence reveal variable survival benefits of living in zoos across mammals. Sci. Rep. **6**, 36361. (10.1038/srep36361) 27819303 PMC5098244

[43] Promislow DEL. 1992 Costs of sexual selection in natural populations of mammals. Proc. R. Soc. Lond. B **247**, 203–210. (10.1098/rspb.1992.0030)

[44] Clutton-Brock TH, Isvaran K. 2007 Sex differences in ageing in natural populations of vertebrates. Proc. R. Soc. B **274**, 3097–3104. (10.1098/rspb.2007.1138) PMC2293943 17939988

[45] Tidière M, Gaillard JM, Müller DWH, Lackey LB, Gimenez O, Clauss M, Lemaître JF. 2015 Does sexual selection shape sex differences in longevity and senescence patterns across vertebrates? A review and new insights from captive ruminants. Evolution **69**, 3123–3140. (10.1111/evo.12801) 26497812

[46] Mitani JC, Gros-Louis J, Manson JH. 1996 Number of males in primate groups: comparative tests of competing hypotheses. Am. J. Primatol **38**, 315–332. (10.1002/(SICI)1098-2345(1996)38:4<315::AID-AJP3>3.0.CO;2-1) 31918481

[47] Rogers LM, Cheeseman CL, Mallinson PJ, Clifton‐Hadley R. 1997 The demography of a high‐density badger (Meles meles) population in the west of England. J. Zool. **242**, 705–728. (10.1111/j.1469-7998.1997.tb05821.x)

[48] Berger J, Gompper ME. 1999 Sex ratios in extant ungulates: products of contemporary predation or past life histories? J. Mammal. **80**, 1084–1113. (10.2307/1383162)

[49] Toïgo C, Gaillard J. 2003 Causes of sex‐biased adult survival in ungulates: sexual size dimorphism, mating tactic or environment harshness? Oikos **101**, 376–384. (10.1034/j.1600-0706.2003.12073.x)

[50] Kappeler PM. 2017 Sex roles and adult sex ratios: insights from mammalian biology and consequences for primate behaviour. Phil. Trans. R. Soc. B **372**, 20160321. (10.1098/rstb.2016.0321) 28760762 PMC5540861

[51] Kappeler PM. 2000 Causes and consequences of unusual sex ratios among lemurs. In Primate males: causes and consequences of variation in group composition (ed. PM Kappeler), pp. 55–63. Cambridge, UK: Cambridge University Press.

[52] Andelman SJ. 1987 10. Ecological and social determinants of cercopithecine mating patterns. In Ecological aspects of social evolution (eds DI Rubenstein, RW Wrangham), pp. 201–216. Princeton, NJ: Princeton University Press. (10.1515/9781400858149.201)

[53] Kappeler PM. 1990 The evolution of sexual size dimorphism in prosimian primates. Am. J. Primatol. **21**, 201–214. (10.1002/ajp.1350210304) 31963975

[54] Kappeler PM, Nunn CL, Vining AQ, Goodman SM. 2019 Evolutionary dynamics of sexual size dimorphism in non-volant mammals following their independent colonization of Madagascar. Sci. Rep. **9**, 1454. (10.1038/s41598-018-36246-x) 30723219 PMC6363729

[55] Kappeler PM, Fichtel C. 2015 Eco-evo-devo of the lemur syndrome: did adaptive behavioral plasticity get canalized in a large primate radiation? Front. Zool. **12**, S15. (10.1186/1742-9994-12-s1-s15) 26816515 PMC4722368

[56] Petty JMA, Drea CM. 2015 Female rule in lemurs is ancestral and hormonally mediated. Sci. Rep. **5**, 9631. (10.1038/srep09631) 25950904 PMC4423346

[57] Kappeler PM, Fichtel C, Radespiel U. 2022 The Island of female power? Intersexual dominance relationships in the lemurs of Madagascar. Front. Ecol. Evol. **10**, 858859. (10.3389/fevo.2022.858859)

[58] Prox L, Fichtel C, Kappeler PM. 2023 Drivers and consequences of female reproductive competition in an egalitarian, sexually monomorphic primate. Behav. Ecol. Sociobiol. **77**, 53. (10.1007/s00265-023-03330-w)

[59] Zehr SM, Roach RG, Haring D, Taylor J, Cameron FH, Yoder AD. 2014 Life history profiles for 27 strepsirrhine primate taxa generated using captive data from the Duke Lemur Center. Sci. Data **1**, 140019. (10.1038/sdata.2014.19) 25977776 PMC4322587

[60] Debyser IWJ. 1995 Prosimian juvenile mortality in zoos and primate centers. Int. J. Primatol. **16**, 889–907. (10.1007/bf02696109)

[61] Silk JB, Brown GR. 2008 Local resource competition and local resource enhancement shape primate birth sex ratios. Proc. R. Soc. B **275**, 1761–1765. (10.1098/rspb.2008.0340) PMC2587797 18445562

[62] Perret M. 1990 Influence of social factors on sex ratio at birth, maternal investment and young survival in a prosimian primate. Behav. Ecol. Sociobiol. **27**, 447–454. (10.1007/bf00164072)

[63] Nunn CL, Pereira ME. 2000 Group histories and offspring sex ratios in ringtailed lemurs (Lemur catta). Behav. Ecol. Sociobiol. **48**, 18–28. (10.1007/s002650000206)

[64] Dias PAD, Montero Domínguez IL, Rangel Negrín A. 2020 Factors influencing infant sex ratio in howler monkeys (Alouatta spp.): a literature review and analysis. Am. J. Phys. Anthropol. **172**, 48–57. (10.1002/ajpa.24035) 32141069

[65] Sussman RW. 1991 Demography and social organization of free‐ranging Lemur catta in the Beza Mahafaly Reserve, Madagascar. Am. J. Phys. Anthropol. **84**, 43–58. (10.1002/ajpa.1330840105)

[66] Richard AF, Dewar RE, Schwartz M, Ratsirarson J. 2002 Life in the slow lane? Demography and life histories of male and female sifaka (Propithecus verreauxi verreauxi). J. Zool. **256**, 421–436. (10.1017/S0952836902000468)

[67] Bayart F, Simmen B. 2005 Demography, range use, and behavior in black lemurs (Eulemur macaco macaco) at Ampasikely, Northwest Madagascar. Am. J. Primatol. **67**, 299–312. (10.1002/ajp.20186) 16287130

[68] Erhart EM, Overdorff DJ. 2008 Population demography and social structure changes in Eulemur fulvus rufus from 1988 to 2003. Am. J. Phys. Anthropol. **136**, 183–193. (10.1002/ajpa.20793) 18257015

[69] Volampeno MSN, Masters JC, Downs CT. 2011 Life history traits, maternal behavior and infant development of blue-eyed black lemurs (Eulemur flavifrons). Am. J. Primatol. **73**, 474–484. (10.1002/ajp.20925) 21254191

[70] Tecot SR, Gerber BD, King SJ, Verdolin JL, Wright PC. 2013 Risky business: sex differences in mortality and dispersal in a polygynous, monomorphic lemur. Behav. Ecol. **24**, 987–996. (10.1093/beheco/art008)

[71] Rolle F, Torti V, Valente D, De Gregorio C, Giacoma C, Von Hardenberg A. 2021 Sex and age-specific survival and life expectancy in a free ranging population of Indri indri (Gmelin, 1788). Eur. Zool. J. **88**, 796–806. (10.1080/24750263.2021.1947398)

[72] Barthold J, Fichtel C, Kappeler P. 2009 What is it going to be? Pattern and potential function of natal coat change in sexually dichromatic redfronted lemurs (Eulemur fulvus rufus). Am. J. Phys. Anthropol. **138**, 1–10. (10.1002/ajpa.20868) 18615575

[73] Ostner J, Kappeler PM. 2004 Male life history and the unusual adult sex ratios of redfronted lemur, Eulemur fulvus rufus, groups. Anim. Behav. **67**, 249–259. (10.1016/j.anbehav.2003.05.012)

[74] Ostner J, Heistermann M. 2003 Endocrine characterization of female reproductive status in wild redfronted lemurs (Eulemur fulvus rufus). Gen. Comp. Endocrinol. **131**, 274–283. (10.1016/s0016-6480(03)00013-3) 12714009

[75] Kappeler PM, Pethig L, Prox L, Fichtel C. 2022 Reproductive senescence in two lemur lineages. Front. Ecol. Evol. **10**, 894344. (10.3389/fevo.2022.894344)

[76] Kappeler PM, Fichtel C. 2012 Female reproductive competition in Eulemur rufifrons: eviction and reproductive restraint in a plurally breeding Malagasy primate. Mol. Ecol. **21**, 685–698. (10.1111/j.1365-294x.2011.05255.x) 21880091

[77] Sibly RM, Collett D, Promislow DEL, Peacock DJ, Harvey PH. 1997 Mortality rates of mammals. J. Zool. **243**, 1–12. (10.1111/j.1469-7998.1997.tb05751.x)

[78] Gerber P, Moisson P, Heistermann M. 2004 Urinary progestogen and estrogen excretion during pregnancy in Eulemur macaco flavifrons, E. rubriventer, and Hapalemur griseus occidentalis. Int. J. Primatol. **25**, 449–463. (10.1023/b:ijop.0000019161.01299.d7)

[79] Shideler SE, Czekala NM, Benirschke K, Lasley BL. 1983 Urinary estrogens during pregnancy of the ruffed lemur (Lemur variegatus). Biol. Reprod. **28**, 963–969. (10.1095/biolreprod28.4.963) 6860749

[80] Heistermann M, Hodges JK. 1995 Endocrine monitoring of the ovarian cycle and pregnancy in the saddle‐back tamarin (Saguinus fuscicollis) by measurement of steroid conjugates in urine. Am. J. Primatol. **35**, 117–127. (10.1002/ajp.1350350204) 31924066

[81] Therneau T. 2023 A package for survival analysis in R. Version 3.5-7. CRAN [Computer software]. See https://cran.r-project.org/package=survival.

[82] Kassambara A, Kosinski M, Biecek P. 2021 Survminer: drawing survival curves using ggplot2. Version 0.4.9. CRAN. [Computer software]. See https://cran.r-project.org/package=survminer.

[83] R Core Team. 2020 R: A Language and Environment for Statistical Computing. Vienna, Austria: R Foundation for Statistical Computing. See https://www.r-project.org/.

[84] Ichino S, Soma T, Miyamoto N, Chatani K, Sato H, Koyama N, Takahata Y. 2015 Lifespan and reproductive senescence in a free-ranging ring-tailed lemur (Lemur catta) population at Berenty, Madagascar. IJFP **86**, 134–139. (10.1159/000368670) 26022309

[85] Rakotonirina H, Kappeler PM, Fichtel C. 2017 Evolution of facial color pattern complexity in lemurs. Sci. Rep. **7**, 15181. (10.1038/s41598-017-15393-7) 29123214 PMC5680244

[86] Watson S, Ward J, Izard K, Stafford D. 1996 An analysis of birth sex ratio bias in captive prosimian species. Am. J. Primatol. **38**, 303–314. (10.1002/(sici)1098-2345(1996)38:43.0.co;2-2) 31918482

[87] Villesen P, Fredsted T. 2006 A new sex identification tool: one primer pair can reliably sex ape and monkey DNA samples. Conserv. Genet. **7**, 455–459. (10.1007/s10592-005-9044-2)

[88] Drea CM. 2011 Endocrine correlates of pregnancy in the ring-tailed lemur (Lemur catta): implications for the masculinization of daughters. Horm. Behav. **59**, 417–427. (10.1016/j.yhbeh.2010.09.011) 20932838

[89] Altmann J, Lynch JW, Nguyen N, Alberts SC, Gesquiere LR. 2004 Life‐history correlates of steroid concentrations in wild peripartum baboons. Am. J. Primatol. **64**, 95–106. (10.1002/ajp.20064) 15356861

[90] Fürtbauer I, Heistermann M, Schülke O, Ostner J. 2012 Brief communication: fecal androgen excretion and fetal sex effects during gestation in wild Assamese macaques (Macaca assamensis). Am. J. Phys. Anthropol. **147**, 334–339. (10.1002/ajpa.21646) 22183710

[91] Kappeler PM. 1997 Intrasexual selection and testis size in strepsirhine primates. Behav. Ecol. **8**, 10–19. (10.1093/beheco/8.1.10)

[92] Dunham AE, Rudolf VHW. 2009 Evolution of sexual size monomorphism: the influence of passive mate guarding. J. Evol. Biol. **22**, 1376–1386. (10.1111/j.1420-9101.2009.01768.x) 19486235

[93] Austad SN, Hoffman JM. 2018 Is antagonistic pleiotropy ubiquitous in aging biology? Evol. Med. Public Health **2018**, 287–294. (10.1093/emph/eoy033) 30524730 PMC6276058

[94] Fromonteil S, Marie-Orleach L, Winkler L, Janicke T. 2023 Sexual selection in females and the evolution of polyandry. PLoS Biol. **21**, e3001916. (10.1371/journal.pbio.3001916) 36626380 PMC9831318

[95] Berger V, Lemaître JF, Dupont P, Allainé D, Gaillard JM, Cohas A. 2016 Age-specific survival in the socially monogamous alpine marmot (Marmota marmota): evidence of senescence. J. Mammal. **97**, 992–1000. (10.1093/jmammal/gyw028)

[96] Regan CE, Medill SA, Poissant J, McLoughlin PD. 2020 Causes and consequences of an unusually male‐biased adult sex ratio in an unmanaged feral horse population. J. Anim. Ecol. **89**, 2909–2921. (10.1111/1365-2656.13349) 32996590

[97] Beirne C, Delahay R, Young A. 2015 Sex differences in senescence: the role of intra-sexual competition in early adulthood. Proc. R. Soc. B **282**, 20151086. (10.1098/rspb.2015.1086) PMC4528560 26156771

[98] Ryan CP, Lee NR, Carba DB, MacIsaac JL, Lin DTS, Atashzay P, Belsky DW, Kobor MS, Kuzawa CW. 2024 Pregnancy is linked to faster epigenetic aging in young women. Proc. Natl Acad. Sci. USA **121**, e2317290121. (10.1073/pnas.2317290121) 38588424 PMC11032455

[99] Pethig L, Ozgul A, Heistermann M, Fichtel C, Kappeler PM. 2025 Data from: Prenatal sex determination illuminates the unusual adult sex ratio of a group-living lemur. Figshare. (10.6084/m9.figshare.26310613)39999891

[100] Pethig L, Ozgul L, Heistermann M, Fichtel C, Kappeler PM. 2025 Supplementary material from: Prenatal sex determination illuminates the unusual adult sex ratio of a group-living lemur. Figshare. (10.6084/m9.figshare.c.7686818)39999891

